# Knowledge, attitude, and associated factor towards cervical cancer prevention among primary and secondary school female teachers in Gondar town, North West Ethiopia, 2022

**DOI:** 10.1186/s12905-023-02498-7

**Published:** 2023-07-10

**Authors:** Birtukan Atena Negash, Netsanet Habtie Bayu, Ashenafi Worku Woretaw

**Affiliations:** Department of Surgical Nursing, School of Nursing, College of Medicine and Health Sciences, University of Gondar, Gondar, Ethiopia

**Keywords:** Cervical cancer, Knowledge, Attitude, Teachers, Gondar, Prevention

## Abstract

**Introduction:**

Cervical cancer is uncontrolled proliferation of cells on the cervix. Worldwide, millions of women suffer from this disease. Cervical cancer can be prevented by increasing awareness and changing negative attitude about the cause and prevention of cervical cancer. The aim of this study was to identify the gap of knowledge, attitude and associated factor about cervical cancer prevention.

**Method:**

Institution-based cross-sectional study was conducted to collect data from 633 female teachers who were working in primary and secondary schools in Gondar town by using a stratified sampling technique. The collected data were checked for any inconsistency, coded, and entered by using EPI INFO version 7 and analyzed by using SPSS version 25. Both Bivariable and multivariable logistic regression analysis was computed to identify the association between the dependent variable with independent variables. Variables having *P*-value < 0.05 were considered statistically significant.

**Result:**

The response rate of this study was 96.4% (610). Of these 38.4% (95% CI; 34.49–42.23) and 56.2% (95% CI; 52.28–60.18) of teachers had good knowledge and positive attitude on cervical cancer prevention respectively. Factors affecting teachers knowledge level were studied language [AOR; 3.9; (1.509–10.122)], Natural Science [AOR 2.9;( 1.128–7.475)], being married [AOR: 0.386; [95% (0.188–0.792)], and heard information from health professionals [AOR; 0.53(0.311–0.925)]. Working in secondary school [AOR; 1.83(1.03–3.25)], have regular menstrual period [AOR; 2.32(1.49–3.62)], no history of abortion, (AOR; 0.45(0.23–0.89), and good knowledge status (AOR, 2.56(1.64–4.00) were significantly associated with positive attitude.

**Conclusion:**

Most of teacher’s knowledge and attitude regarding to cervical cancer prevention were poor. Being married, the field of study, and natural science, heard information from health professionals were factors associated with knowledge. Working in secondary school, having regular menstrual period, no history of abortion, and good knowledge were factors associated attitude regarding to cervical cancer prevention. Therefore, enhancing health promotion through mass media and established counseling program with reproductive health is important.

## Background

Cervical cancer is a malignant of neoplasm arising from the cervix. It may be completely asymptomatic at early stages [1]. It is the fourth most common cancer leading cause of mortality in women of reproductive age group worldwide mainly in developing countries [2–4]. It is a global pandemic disease affecting both developed and developing countries and rapidly increasing in low and middle-income countries [5]. Mostly two types of virus that cause cervical cancer in women are HPV16 and HPV18, which are responsible for approximately 70% of cervical cancers worldwide. Early starting sexual intercourse, multiple sexual partners of spouse, sexual transmitted infection, short gap between birth and immune suppression are the main risk factors for development of cervical cancer [6]. The estimated incidence of cancer related death among women which result from cervical cancer in Sub-Central America, South-central Asia, and Malezya [7]. International Agency for Research on Cancer (IARC) and WHO estimated that 85% of the worldwide deaths from cervical cancer occur in developing countries, higher than 18 times of death rate which compared with developed countries [7, 8] In Africa, a population of 35 per 100,000 new cases, and 23 death occurs per 100,000 women every year and the prevalence of cervical cancer is very high in Sub-Saharan Africa [9]. A systematic review study in Ethiopia showed that the prevalence of late stage of cervical cancer among cervical cancer patient was 56.8% due to the scarcity of information, cost of service,and fear of screening procedure [10].

Some evidence in Iraq, Saudi Arabia, and India showed that most of teachers have poor knowledge and negative attitude regards to cervical cancer prevention a result from lack of awareness, and adopting unhealthy lifestyle which factors influencing cervical cancer prevention [11–13]. The study conducted in Nigeria, Akwa, majority of teachers have inadequate knowledge and unfavorable attitude about early detection of cervical cancer, and other methods of cervical cancer prevention [14]. According to University of Ibadan study even if the majority of female teachers has positive attitude but the knowledge status of many teachers was poor about cervical cancer prevention [15].

Another studies was conducted in Nigeria, surulere and Bagdad, indicates that most of the female teachers had heard about cervical cancer but the knowledge and attitude status was very low which results from shortage of formal education and counseling regarding to cervical cancer prevention [16, 17]. In Kenya, the majority of participants did not know cervical cancer prevention due to lack of enough information, and fear of screening and side effects [18]. A study conducted in Hawassa, Ethiopia revealed that only 27.2% were knowledgeable [19].

Many studies also recommended primary prevention which involves the prevention of HPV infection achieved by increasing knowledge, enhancing positive attitude, and use biological mechanisms of prevention, including HPV vaccination, and abstinence from sexual exposure [20]. In secondary prevention, periodic cervical cancer screening helps the early diagnosis and treatment of the lesion [21].

So, improving teachers knowledge and attitude of cervical cancer prevention by enhancing clear understanding, and enough awareness about early detection in developing countries is important [11, 22]. Many factors are implicated including lack of information, negative attitudes, cost of service, fear of the procedure, and the fact about cervical cancer prevention methods [23, 24]. Even though there is a high magnitude of cervical cancer, there was a limited study available on the knowledge as well as factors associated with cervical cancer prevention but did not addressed attitude of female teachers in Ethiopia. Still now Knowledge and attitude are crucial to the prevention of cervical cancer on female teachers. If the teachers’ have good knowledge & positive attitude towards cervical cancer prevention they also create awareness to their students in addition to preventing themselves about the causes, risk factors and prevention strategies by establish club and promote health education in the schools. So the main aim of this study was to assessing level of female teachers’ knowledge, attitude and associate with cervical cancer.

## Method and materials

### Study design and period

The institutional-based cross-sectional study design was conducted from May 15-June 15, North, west, Ethiopia, 2022.

### Study setting

The study was conducted in primary and secondary schools in Gondar town. Gondar is one of the ancient cities which were established by emperor Fasilades in 1632 Ethiopian calendar, it contains seven ancient heritages that are found in the central Gondar zone of Amhara Regional State. Gondar is located 727 km Northwest of Addis Ababa and 173 km from the capital city of Amhara Regional State of Bahirdar to the North. The geographical location is 120 3’N latitude and 370 28’E latitude [25, 26]. According to the 2007 Ethiopian census report, Gondar town has a total population of 206,987, and more than half (108,902) of them were female [27]. The town is divided into six administrative sub cities. According to Gondar town education administration office, Gondar town has 60 primary and 20 secondary schools. Of the total school, 20 primary and 5 secondary schools are private schools, and 40 primary and 15 secondary are governmental schools. Of those private and governmental schools, 1246 female and 672 male teachers are working in the primary schools, and 318 female and 523 male teachers working in secondary schools.

### Source population and study population

All female teachers who were working in primary and secondary schools in Gondar town administration was a source of population.

All female teachers who were working in selected primary and secondary schools during the data collection period was the study population.

### Inclusion and exclusion criteria

All-female teachers who work in selected primary and secondary schools during the data collection period were included in study. Female teachers who work part-time in both governmental and private schools are excluded from the study.

### Sample size determination and calculation

For the dependent variable: the sample size was determined by using single population proportion formula and the proportion was taken from previous study conducted in Hawassa town [19]. The knowledge of the respondents was 27.2% by considering 95% confidence interval (CI) and 5% marginal error, sample size was calculated as follows. By adding 10% non-response rate the final sample size was 334 female teachers.

Whereas dependent variable of attitude cannot study in Ethiopia since I have taken proportion (50%) calculated sample size was 384

$${\varvec{n}}=\frac{({\varvec{Z}}{\varvec{a}}/2)2{\varvec{P}}(1-{\varvec{P}})}{{\varvec{d}}2}$$

where: n = required sample size.

Z = the standard normal deviation at 95% confidence interval; = 1.96.

P = Assumed proportion of nurse 27.2%( 0.272%).d = margin of error that can be tolerated, 5% (0.05)

1-p = proportion of population that do not possess the character of interest.

### Sample size for the second objective (Table 1)

**Table 1:** Sample size determination for the second objective

Factors associated with knowledge	Assumption	% of cases among exposed	% of cases among unexposed	COR	Initial sample size	Final sample size after adding 10%contingency
Age	Power = 80 CI = 95% Ratio 1:1	42.37%	23.37%	2.410	212	233
Being ever having pregnancy		33.57%	18.18%	2.274	278	305

$$Therefore\boldsymbol{ }\,{\varvec{n}}=\frac{\left(\frac{{\varvec{Z}}{\varvec{a}}}{2}\right)2*{\varvec{P}}(1-{\varvec{P}})}{{\varvec{d}}2}=\frac{\left(1.96\right)2*0.272(1-0.272)}{\left(0.05\right)2}=304$$

For the independent variable female teacher's cervical cancer prevention age with (COR 2.410), being ever having pregnancy with (COR 2.274) was significantly associated with teacher’s knowledge of cervical cancer prevention in multivariable analysis respectively.

By adding design effect 1.5 the final sample size was 576.

By adding 10% non-response rate the final sample size was 633.

### Sampling technique and procedures

**Fig. 1:**
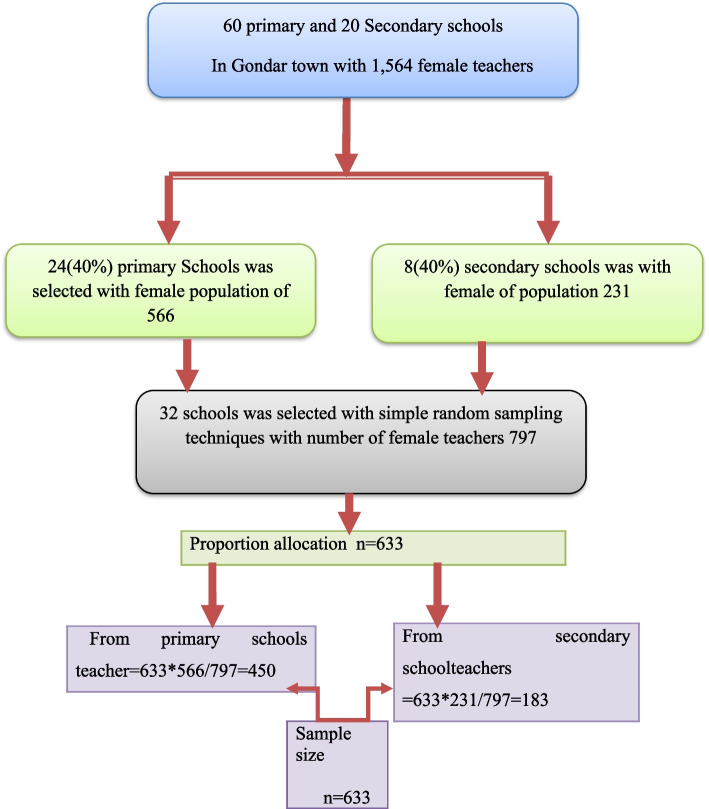
Schematic presentation of sampling procedure on knowledge, attitude, and associated factor towards cervical cancer prevention and among female teachers working at primary and secondary school in Gondar town, Northwest Ethiopia 2022

**Fig. 2:**
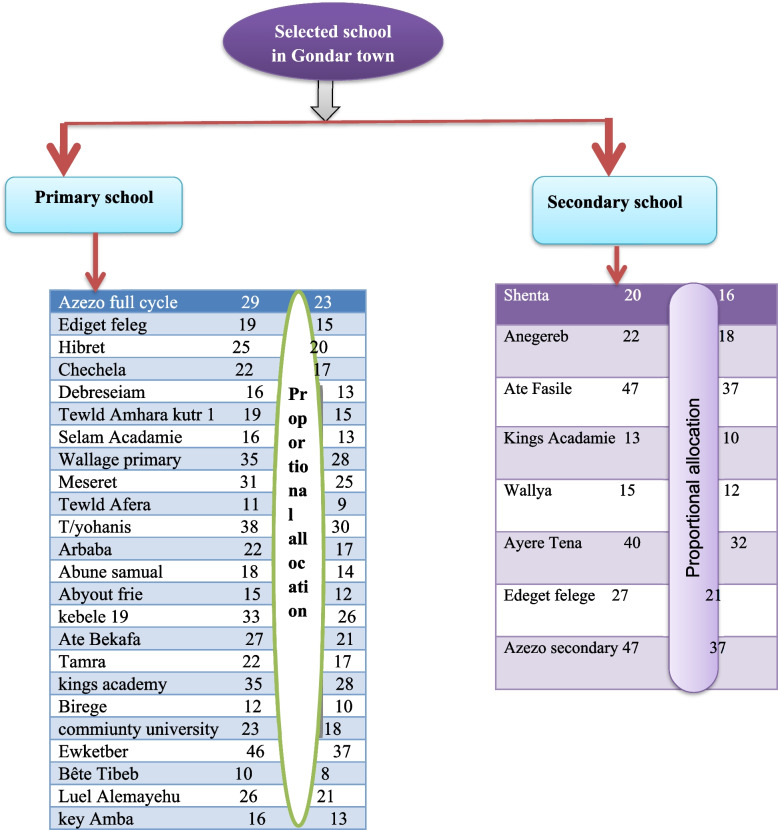
Proportional allocation of sampling in each school among female teachers working at primary and secondary schools in Gondar town, Northwest Ethiopia 2022

Firstly, list all of the schools were obtaing from Gondar twon education administration office.Then schools were groupd in to two primary and secondary schools. Which made selected fourty percent of 60 primary and fourty percent of 20 secondary schools was 24 and 8 respectively school (Fig. 1).the list of 24 primarly and 8 secondary schools selected by lottery method.who works 566 female teachers in 24 primary and 231 female techars in 8 secondary schools. Then, list selected schools and the sample size was selected 450 from the primary and 183 from the scondary schools with proportional allocation formula (Fig. 2). Allocating sampling proportional formula to the total population of each stratum using this formula.

Where n = total sample size to be selected, $\mathrm{n}=\frac{\mathrm{n}*\mathrm{Ni}}{\mathrm{N}}$ N = total population Ni = total population of each strata and n = sample size from each stratum.

The final sample size was 633 female techers who work in selected schools in Gondar town which involves in the study. Finaly simple random sampling technique were used for the selected of female teachers in each schools.

### Data collection tools and procedure

A structured self-administered questionnaire adapted from previous literature and modified from other similar studies was used to collect the data [14, 17, 23]. The questionnaire was prepared in the form of English and translated to Amharic and back to English to check whether it is consistent or not. The questionnaire had four sections. The 1^st^ section was composed of socio-demographic information about the participants including age, marital status, religion, monthly income, level of education, field of working, working, husband education and source of information. The 2^nd^section was information on the reproductive and lifestyle factor of participants. The 3^rd^ section was composed of 15 questions that assess the knowledge of teachers. The 4^th^ section was composed of 18 questions to assess the attitude of teachers the response set is a 3-

Point Likert scale that consists of responses such as (‘‘Agree’’ = 1 Disagree’’ = 2 and ‘‘undecided’ = 3’ during data collection. It was also recoded into 1 and 0. A score of 1 is interpreted as a correct response; while 0 is applied for the incorrect responses.

Five Bsc nurses for data collection were recruited for data collector and three MSc nurses were recruited as a supervisor.

#### Operational definition

##### Knowledge

Is the fact or condition of knowing cervical cancer prevention well that is obtained through experience and training [28].

##### Good knowledge

Those teachers who scored median and above the knowledge questions were considered as Good knowledge.

##### Poor knowledge

Those teachers who scored below the median of the knowledge questions were considered as poor knowledge.

##### Attitude

Pattern of teachers mental views towards cervical cancer prevention characteristics [29].

##### Positive attitude

Those teachers who score median and above of the attitude questions are considered as positive attitude.

##### Negative attitude

Those teachers who score below the median of the attitude questions are considered as negative attitude.

### Data quality control

Training for data collectors and supervisors was prepared and given by the principal investigator two days prior to the beginning of data collection regarding to the objectives of the study, the data collection approach, the contents and the relevance of the study, the confidentiality of the information, as well as the rights of participants. Before starting data collection, a pretest was conducted on five percent of school teachers at keble10, and Hidasy Tseda primary and secondary schools. During pretesting the tool was checked for its clarity, simplicity, understandability, completeness consistency, and coherency. Appropriate measures and corrections were taken on time for completeness and accuracy before the beginning of data collection. Finally, the questionnaire was delivered to the study participants, and data collection was done by five trained BSc nurse professionals. The supervisors strictly supervised the data collection process and provide onsite advice and feedback to the data collectors as required on regular basis. Daily exchange of information between the principal investigator and supervisors was undertaken by face-to-face and telephone.

The collected data was examined for its completeness & accuracy during data collection to ensure the validity of the questionnaire. Face validity was done by advisors and the internal consistency was checked by computing Cronbach’s α with the value of 0.7% from the pretest data. The tests were shows 0.73 and 0.79 for knowledge and attitude, respectively.

### Data processing and analysis

After data was collected from the selected schools, filled questionnaires were checked manually for correctness and completeness daily. After checking all questionnaires, Data was entered into EPI info version 7.0, and the data was exported to statistical package for social science (SPSS) version 25.0 software for analysis. Binary logistic regression was employed. In the bivariable analysis, independent variables with *p*-value less than 0.25was entered into multivariable analysis to control the effect of confounding variables. Variables having a *p*-value < 0.05 and 95% CI in multivariable analysis were used to interpret the association between dependent variable and independent variable. Multicollinearity was checked by linear regression using the variance inflation factor. Model adequacy was checked by using Hosmer and Lemeshow so the model indicates a good fit which indicates 0.95 and 0.605 for knowledge and attitude respectively.

## Results

### Socio-demographic characteristics of the participant

A total of six hundred (633) teachers were selected from primary and secondary schools in Gondar town participated in the study. The response rate was 96.4%. The median age of the participants was 36 were within the range between 22 and 59 years. (, Four hundred fourteen (67.9%) were married, among the being married participants 276 (66.7%) had higher husbands educational status (Table 2).

**Table 2:** Socio-demographic characteristics of the respondents on knowledge and attitude of cervical cancer prevention among primary and secondary school female teachers in Gondar town Northwest Amhara, Gondar, 2022 (*n* = 610)

**Variables**	**Response**	**Frequency (*N*** **= 610)**	**Percent (%)**
Age	20–30	120	19.7
	31–40	357	58.5
	41–50	91	14.9
	> 50	42	6.9
Religion	Orthodox	531	87
	Muslim	43	7.1
	Protestant	36	5.9
Marital status	Single	139	22.8
	Married	414	67.9
	Divorced	49	8
	Widowed	8	1.3
Working school	Primary school	427	70
	Secondary school	183	30
Level of education	Certificate	16	2.6
	Diploma	250	41
	Degree	286	46.9
	MSC Degree	58	9.5
Monthly income	3000–4999	81	13.3
	5000–8000	262	43
	> 8000	267	43.8
Field of study	Language	173	28.4
	Social Science	173	28.4
	Natural Science	208	34.1
	Art	56	9.2
**Variable**	**Response**	**Frequency**	**Percentage (%)**
Husband education	No regular education	17	4.1
	Primary education	37	8.9
	Secondary education	84	20.3
	Higher education	276	66.7
Source of information	Health professional	498	81.6
	from radio/TV	105	17.2
	Colleague, social and Magazines	7	1.1

### Reproductive and lifestyle characteristics

Out of the participants, 51 (11.3%) participants had the birth gap between their children less than 2 years. Of the abortion history of the participants 54 (12%) had history of one abortion, and 15 (3.3%) were had two and more history of abortion, 386(63.3%) were had regular menstrual periods (Table 3).

**Table 3:** Reproductive and lifestyle factors of the respondents on knowledge and attitude of cervical cancer prevention in Gondar town Northwest Amhara, Gondar, 2022 (*n* = 610)

Variable	Response	Frequency *N* = (610)	Percentage (%)
Age of first menstrual period	< 15	385	63.1
	> 15	225	36.9
Pattern of menses	Regular	386	63.3
	Sometimes Irregular	194	31.8
	Always irregular	30	4.9
Age First sexual intercourse	< 20	130	21.3
	≥ 20	480	78.7
sexual partner	One	559	91.6
	Two and above	51	8.4
History of pregnancy	Not getting pregnancy	160	26.2
	Having pregnancy	450	73.8
History of abortion (*n* = 450)	Not Having abortion	381	84.7
	One Abortion	54	12
	Two and more abortion	15	3
Age at first pregnancy (*n* = 450)	< 20	18	4
	20–30	377	83.8
	≥ 30	55	12.2
Birth gap between babies (*n* = 450)	< 2 years	51	11.3
	≥ 2 years	301	66.9
	Only one child	98	21.8
Breast feeding (*n* = 450)	Yes	399	88.7
	No	51	3
Duration of breast feed (*n* = 399)	< 2 years	126	31.6
	≥ 2 years	273	68.4
Use of birth control pills	use	341	55.9
	never use	269	44.1
History of STI	Yes	27	4.4
	No	583	95.6
Getting treatment of STI	Treated	23	85.2
	Not treated	4	14

### Knowledge of teachers on cervical cancer prevention

**Fig. 3:**
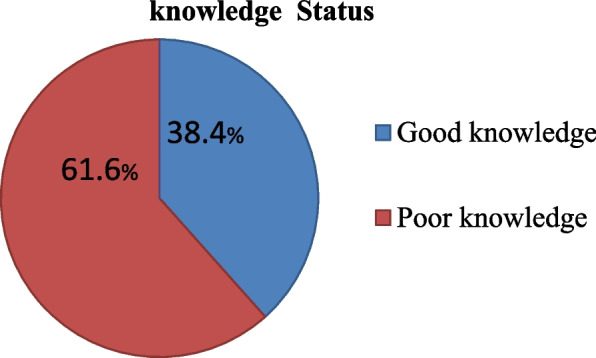
Knowledge of participants towards cervical cancer prevention among primary and secondary school female teachers North West Ethiopia, 2022

From fifteen knowledge assessment questions regarding to cervical cancer prevention (38.4%) [95% CI; 34.49–42.23] of the teachers had good knowledge (Fig. 3). The majority of participants 498(81.6%) were heard information from health professionals. About 536 (87.9%) and 514 (84.3%) of the study participants reported cervical cancer is preventable and curable respectively. More than half participants 435 (71.3%) were did not aware about HPV infection (Table 4).

**Table 4:** Frequency distributions of knowledge regarding cervical cancer prevention among female primary and secondary school teachers in Gondar town Northwest Ethiopia, 2022 (*N* = 610)

Statement about cervical cancer prevention	Response	Frequency	Percentage (%)
Have you ever heard about cervical cancer	Yes	610	100%
	No	0	0%
causative agent of cervical cancer	Virus	208	34.1%
	Bacteria	143	23.4%
	Fungi	143	23.4%
	parasite	14	2.3%
	I don't Know	102	2.3%
Do you know the risk factors of cervical cancer	Yes	240	39.3%
	No	370	60.7%
Do you know HPV infection	Yes	175	28.7%
	No	435	71.3%
Do you know about the symptoms and signs of cervical cancer	Yes	141	23.1%
	No	469	76.9%
Cervical cancer is preventable	Yes	536	87.9%
	No	74	12.1%
Can prevent cervical cancer by avoid multiple sexual partner	Yes	214	35.1%
	No	396	64.9%
Can prevent cervical cancer by screening	Yes	114	18.7%
	No	496	81.3%
Do you know vaccination of cervical cancer	Yes	166	27.2%
	No	444	72.8%
Is cervical cancer curable	Yes	514	84.3%
	No	96	15.7%
Cervical can be cured at early stage	Yes	369	60.5%
	No	241	39.5%
Did you know cervical cancer screening	Yes	339	55.6
	No	271	44.4%
screening is used to early detection of cervical cancer	Yes	184	30.2%
	No	426	69.8%
Screening is important to sexually active women	Yes	145	23.8%
	No	465	76.2%
DO you know cervical cancer screening interval	Yes	126	20.7%
	No	484	79.3%

### Attitude of teachers regarding cervical cancer prevention

**Fig. 4:**
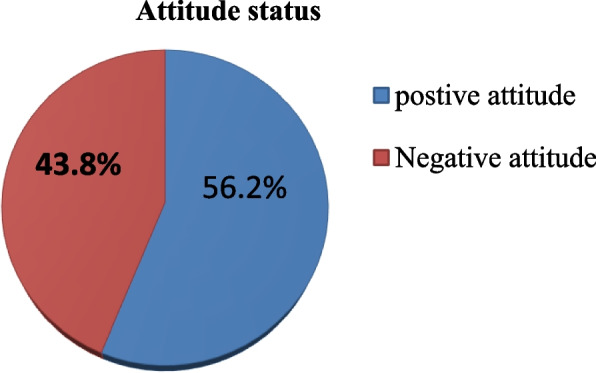
Attitude of participants towards cervical cancer prevention among primary and secondary school female teachers North West Ethiopia, 2022

From the total of 610 participants, this finding showed that 56.2% (95% CI; 52.2, 60.1) of the respondent had positive attitude regarding to cervical cancer prevention (Fig. 4). Among the total participants, 499 (81.8%) disagree with the idea of cervical cancer screening is unnecessary if the person has a symptom, and 294 (48.2%) were agree with the idea of procedure for cervical cancer screening is pain full. One hundred sixteen (19%) participants agree with the idea of believe it is shameful and embarrassing to undergo cervical cancer treatment, 176 (28.9%) participants agree with believe if know my status of cervical cancer I will die before time (Table 5).

**Table 5:** Teacher's Attitude regarding cervical cancer prevention in Gondar Town North west Ethiopia, 2022 (*N* = 610)

Statement of Attitude	Agree		Neutral		Disagree	
	N	%	N	%	N	%
I am healthy and cannot develop cervical cancer	95	15.6	77	12.6	438	71.8
Believe cervical cancer screening is unnecessary if the person has symptom	69	11.3	42	6.9	499	81.8
I feel that cervical cancer is the disease of the elderly only	78	12.8	61	10	471	77.2
One’s sexual lifestyle does predispose to cervical cancer	74	12	78	12.8	458	75.1
Do you think cervical cancer is a punishment from the God	70	11.5	46	7.5	494	81
Cervical cancer is caused by witches and wizards	49	8	53	8.7	508	83.3
I believe it is shameful and embarrassing to undergo cervical cancer treatment	116	19	56	9.2	438	71.8
Do you think going for screening is lack of faith and belief in God	98	16.1	56	9.2	456	74.8
The procedure for cervical cancer screening is painful	294	48.2	91	14.9	225	36.9
If I know my status of cervical cancer I will die before time	176	28.9	75	12.3	359	58.9
I will be stigmatized by my spouse if test positive to cervical cancer	176	28.9	82	13.4	352	57.7
I think Cervical cancer cannot be prevented	101	16.6	58	9.5	451	73.9
Only God can prevent cervical cancer	125	20.5	64	10.5	421	69.9
Regular screening for cervical cancer can reduce the risk of cervical cancer	403	66.1	40	66	167	27.4
Regular washing of the genitals with soap can help prevent cervical cancer	301	49.3	80	13.1	229	37.5
I think to eat fruit and vegetable to prevent cervical cancer	366	60	65	10.7	179	29.3
I think taking action to avoid unprotected sex can prevent cervical cancer	438	71.8	28	4.6	144	23.6
I think I will go for treatment when I see any of STI symptom	466	76	25	4.1	119	19.5

### Factors associated with knowledge and attitude of cervical cancer prevention

According to bi-variable logistic regression analysis, among twenty-two independent variables, twelve variables were found in a *P*-value of < 0.25. The variables significantly associated with Knowledge of cervical cancer prevention were entered into the multivariable analysis. According to the multivariable logistic regression analysis, the participants who had fields of study language were about 4 times [AOR = 3.9; [95%CI (1.509–10.122), and Natural science were about 3 times [AOR = 2.9(1.128–7.475) more likely to have good knowledge than art. Being married participants were 61.4% less like (AOR: 0.38; [95% (0.188–0.792,)] to have good knowledge compared to others (widowed and divorced). heard information from health professionals were 46.3% less like (AOR 0.537(0.311–0.925) to have good knowledge compared as heard information from radio/TV, colleague, and social media (Table 6).

**Table 6:** Bivariable and multivariable analysis of factors associated with Knowledge of primary and secondary school female teachers regarding cervical cancer prevention in Gondar town North West Ethiopia, 2022 (*N* = 610)

Variable		Knowledge of teachers		COR (95%CI)	AOR (95% CI)	*P*-value
		Good	Poor			
Age	21–30	42	78	1		
	31–40	130	227	1.64(0.690–1.1639)	0.662(0.270–1.620)	0.36
	41–50	40	51	1.457(0.83–2.54)	0.647(0.238–1.761)	0.39
	> 50	22	20	2.043(1.002–4.165)	0.854(0.273–2.820)	0.82
Marital status	Others	27	30	1	1	
	Single	45	94	0.532(0.283–0998)	0.775(0.235–2.551)	0.67
	Married	162	252	0.714(0.410–1.246)	0.386(0.188–0.792)	0.009**
Level of education	Diploma and below	82	184	1	1.732(0.165–18.172)	0.647
	Degree	124	162	1.718(1.210–2.437)	0.936(0.529–1.657)	0.82
	Master	28	30	2.094(1.176–3.729)	1.044(0.404–2.698)	0.92
Monthly income	3000–4999	22	59	1		
	5000–8000	103	159	1.737(1.004–3.008)	2.048(0.916–4.576)	0.081
	> 8000	109	158	1.850(1.071–3.192	1.839(0.754–4.485)	0.18
Field of the study	Art	14	42	1		
	Language	75	98	2.296(1.168–4.511)	3.908(1.509–10.122)	0.005**
	Social Science	59	114	1.553(0.785–3.070)	2.210(0.839–5.821)	0.10
	Natural Science	86	122	2.115(1.088–4.111)	2.904(1.128–7.475)	0.027**
School you teach	Primary	142	285	1		
	Secondary	92	91	2.029(1.426–2.888)	1.646(0.916–2.957)	0.095
Age of First menstrual	< 15	135	250	1		
	> 15	99	126	1.455(1.039–2.037)	1.154(0.737–1.805)	0.53
Sexual partner	Two and above	17	56	1	1	
	One	217	320	0.48(0.253–0.791)	2.308(0.978–5.50)	0.056
Age first pregnancy	< 20	7	11	1	1	
	20–30	143	234	0.960(0.364–2.534)	0.703(0.234–2.115)	0.53
	> 30	32	23	2.186(0.736–6.494)	1.717(0.495–5.951)	0.39
Use of birth control pills	Use	122	219	1	1	
	Never use	112	157	1.281(0.922–1.7781)	0.969(0.607–1.546)	0.89
Duration of breastfeeding	< 2 years	44	82	1	1	
	≥ 2 years	144	159	1.336(0.862–2.071)	1.232(0.763–1.991)	0.39
Source of information	Radio/TV, Social media	178	320	1	1	
	Health professional	56	56	0.556(0.368–0.841)	0.537(0.311–0.925)	0.025**

Note

Others: Divorced, Widowed

^***^ *P* value < 0.001 strongly assocaited

^**^ *p* value < 0.01weak associated

^*^ *p* value < 0.05 statistical significance

Teachers who work in secondary school were about 2 times [AOR; 1.83(1.03–3.25)] more likely have positive attitudes than teachers who work in primary schools. Teachers who had regular menstrual period were 2 times [(AOR; 2.32(1.49–3.62)] More likely to have positive attitudes than teachers who had irregular menstrual period. Teachers who had no history of abortion were 55% less like [(AOR; 0.45(0.23–0.89)] to have positive attitude compared to teachers who have history of abortion. Teachers who had good knowledge were 2 times [(AOR (2.56(1.680–4.050)] have positive attitude as compared to teachers who had poor knowledge regarding to cervical cancer prevention (Table 7).

**Table 7:** Factors associated with attitude regarding cervical cancer prevention among primary and secondary school Female teachers in Gondar Town, North West Ethiopia, 2022 (*N* = 610)

Variable		Attitude		COR (95%CI)	AOR (95% CI)	*P*-value
		Positive	Negative			
The school /working Level of education	Primary	228	199	1	1	
	Secondary	115	68	1.476(1.035–2.105)	1.83(1.03–3.25)	0.038**
	Diploma and below	146	120	1	1	
	BSc Degree	165	121	1.12(0.80–642)	0.96(0.17–5.29)	0.96
	MSc degree	32	26	1.01(0.65–6.39)	0.44(0.06–2.84)	0.39
Field of study	Art	25	31	1	1	
	Language	105	68	1.915(1.042–3.520)	0.91(0.52–1.61)	0.76
	Social Science	92	81	1.408(0.769–2.581)	0.98(0.58–1.67)	0.95
	Natural Science	121	87	1.725(0.952–3.125)	0.76(0.35–1.67)	0.50
Pattern of menstrual period	Irregular	232	154	1	1	
	Regular	111	113	1.534(1.101–2.137)	2.32(1.49–3.62)	0.000***
History of abortion	Have abortion	37	17	1	1	
	No abortion	208	173	1.810(0.98–3.32)	0.45(0.23–0.89)	0.022**
Use birth control pills	Never use	163	106	1	1	
	I use	180	161	0.727(0.526–1.005)	0.83(0.54–1.27)	0.40
Knowledge	Poor	185	191	1	1	
	Good	158	76	2.146(1.527–3.016)	2.56(1.64–4.00)	0.000***

Note

*Abbreviations*: *AOR* Adjusted odds ratio, *COR* Crude odds ratio, *CI* Confidence interval

^***^ *P* value < 0.001 strongly associated

^**^ *p* value < 0.01weakly associated

^*^ *p* value < 0.05 statistical significance

## Discussion

This finding showed that 38.4% of teachers had good knowledge with the confidence interval of [95% (34.49–42.23)]. This study is lower than those study done, in Srilanka 50.5% [30], Riyadh in Saudi Arabia 43% [6], Surulere 73.7% [17], Bangalorine in India 66% [31], Mushin in Nigeria 100% [23] taraba 92.5% [32] and Ibadan 79.9% [15]. This discrepancy might be due to setting, and health education strategies in countries. The discrepancy of the study conducted in Bangalorine and Tarab the data was collected after giving training regarding to cervical cancer prevention since training might be increasing the knowledge status of teachers. In Riyadh and Surulere the study was conducted in only secondary school teachers those teachers might have higher educational status and experience to access more information there might have more knowledge status.

But this finding is higher than study was conducted in Iraq 32.4% [11],Bagdad 10% [16], Nepal 12.7% [33], Ambear state of Nigeria 14% [34], legose 18.1% [35], Akwa 28.8% [14], Hawassa Ethiopia 27.2% [19]. Those variations may be due to the data collection methods. Some of the above mentoid countries used interview data collection method therefore the interview data collection method might be give limited information about sensitive issues, time-consuming for response, and tiresome so the participants might be to give low information about cervical cancer prevention [36]. When we compare the study conducted in Hawassa, Ethiopia includes only the primary school teachers but this study involves the secondary school teachers. Most of secondary school teachers might have a Degree and MSc level of educational status and experience to access information it might be increased their knowledge status.

Regarding to attitude, this finding revealed that 56.2% of had positive attitude with a confidence interval of [95% (52.2–60.1) towards cervical cancer prevention. This study findings is lower than study were conducted in Iraq 76.6% [11], Ibadan 86.4% [15], Legos 67.8% [35] surulere 94.1% [17]. This possible reason might be due to individual perception (view) and those countries to give traing for the study participants before data collection period traing might be change negative attitude. The result of this study showed that higher than Indian 28.7% [37], Kenya 40% [18]. This difference might be due to the socio-cultural difference of study participants, health care delivery policy, and health education strategies in each country.

The finding of this study revealed that the fields of study were one of the factors significantly associated with teachers’ knowledge of cervical cancer prevention. Teachers who study language (Amharic and English) about 3 times [95% CI (1.509–10.122)] and natural science about 3 times [95% CI (1.128–7.475) times more likely to have good knowledge compared with art. A possible justification of this result the teachers who studied language might be easily understanding the message of different books and magazine, health professionals, mass media, and social media, and have less language barrier for communication with health professionals [38]. The other field of study is natural science. Teachers who studied natural science might have information about health related topics, disease, reproductive system and hormonal changes in the body like the department of biology, it might be getting more information about cervical cancer and it is prevention. The Study participants who had being married were 61.4% less likely (AOR: 0.386; [95% (0.188–0.792)] to have good knowledge compared to others (widowed, and divorced).

The reason of this result might be teachers who had being married have different responsibilities in their family, home activities like baby care and food preparation in addition to teaching responsibilities and might have no enough time for read, attend training and different workshop, so they might not have updated information regarding cervical cancer prevention.

The other socio-demographic related factor which significantly associated with teachers’ knowledge of cervical cancer prevention in this study is source of Information. The Study participants who heard information about cervical cancer from health professionals were 46.3% less likely (AOR 0.537(0.311–0.925) to have good knowledge about cervical cancer prevention compared with heard from radio/TV, colleague, and social media. The possible reason of this result might be teachers obtain information from the health professionals is limited due to limited education and counseling program. Another reason might be lack of health professionals and governmental agencies for established health education and traing program in the schools as community service. But information from radio/TV, Colleague, and social media gives short, precise, and repeated day-to-day information and more address within a short period of time. This evidence is supported by study conducted in Arab Emirates [39]. The findings of this study revealed that teachers who have good knowledge towards cervical cancer prevention 2.56 [95%( 1.64–4.00)] times more likely to have positive attitude towards cervical cancer prevention compared with poor knowledge. This possible reason might be teachers who have good knowledge might have enough information regarding to cervical cancer, the benefits of cervical cancer prevention than treatment and it might be change negative attitude to positive attitude of cervical cancer prevention. This evidence is supported by Taraba [32]. Another factors which associated with attitude is history of abortion, no history of abortion 55% less likely [AOR 0.45 (0.23–0.89) to have positive attitude towards cervical cancer prevention compared with who have history of abortion. The possible justification of this result might be those who had no history of abortion have not obtaining opportunity of counseling and information from health professionals about post-abortion care, complication of abortion, risk of abortion on cervical cancer, and cervical cancer prevention since it might be affect the status of positive attitude compared with teachers had history of abortion [40].

Other variables, those teachers who have regular menstrual period 2 times with (AOR; 2.32 (1.49–3.62) time more likely to have positive attitude regard to cervical cancer prevention compared with teachers who have irregular menstrual period. The reason of this finding might be abnormal (irregular) menstrual period has major impact women quality of life with mood change, depression, pain, headache, and left from workplace due to this reason teachers who have irregular menstrual decrease the need of obtain information and screening services but regular menstrual period not affect the quality of life, mood change, not cause the above problem might lead to positive attitude this evidence supported by Chicago.

### Limitations

Due to the time limitation qualitative approach was not done.

### Conclusion and recommendations

This finding showed that Knowledge and attitude regarding to cervical cancer prevention was poor. Being married, field of study language and natural science, and sources of information from health professionals were factors associated with teachers’ knowledge. Working in secondary school, having regular menstrual period, no history of abortion and knowledge were factor associated with attitude towards cervical cancer prevention. Therefore, health promotion, counseling integrated with reproductive health education through mass media in simple and understanding way regarding to cervical cancer prevention should be important.

## Data Availability

The datasets used/or analyzed during the current study is available from the corresponding author on reasonable request.

## Abbreviations

AOR: Adjusted Odds Ratio
CC: Cervical Cancer
CI: Confidence Interval
HPV: Human Papilloma Virus
IQR: Inter Quartile Range
IRA: Institutional Revised Board
LMICS: Law and middle-income Countries
SPSS: Statically Package of Social Science
SSA: Sub Saharan Africa
STD: Sexual Transmitted Disease
STI: Sexual Transmitted Infection
WHO: World Health Organization
